# Supplementary material for: Phenotypic Spectrum and Molecular Findings in 17 ATR-X Syndrome Italian Patients: Some New Insights
Source: Genes (Basel). 2022 Oct 4;13(10):1792. doi: 10.3390/genes13101792 (PMC9601810; doi:10.3390/genes13101792)
Supplement: Supplementary file 1 [file genes-13-01792-s001.zip › genes-1944965-supplementary.pdf]

**Supplementary Table S1**

Distribution of the mutations among the different protein domains

| *Involved domain* | *N. of different mutations* | *N. of independent cases* | *%* |
|---|---|---|---|
| ***ADD*** | 3 | 7 | 50 |
| ***Helicase*** | 4 | 4 | 28.5 |
| ***Others*** | 3 | 3 | 21.5 |
